# Impact of Ethical Ideologies on Students’ Attitude toward Animals—A Pakistani Perspective

**DOI:** 10.3390/ani13050927

**Published:** 2023-03-03

**Authors:** Asiya Khalid, Pim Martens, Aliya Khalid

**Affiliations:** 1 Department of Applied Psychology, National Institute of Modern Languages, Islamabad 44000, Pakistan; 2 University College Venlo, Maastricht University, 6211 Maastricht, The Netherlands; 3 College of Graduate and Postdoctoral Studies, University of Saskatchewan, Saskatoon, SK S7N 5C9, Canada

**Keywords:** ethical ideologies, attitude toward animals, animal welfare, cultural differences, undergraduate students

## Abstract

**Simple Summary:**

Ethical ideologies can have a strong impact on the moral decision making of students. They may also impact their reasoning regarding different social concerns including animal welfare. There are two types of ethical ideologies, including idealistic ideology (a view that universal principles exist which can be followed while making moral decisions) and relativistic ideology (a view that ethical decision making varies from situation to situation). For students, future decision making and concern for animal welfare can be influenced by their ethical perspectives, and to promote a positive change, it is imperative to promote understanding and awareness while students are still in the current educational structure. Hence, the research study explored the relationship between ethical ideologies (idealism and relativism), attitude toward animals and concern for animal welfare among students. Results showed that both relativistic and idealistic ideologies were associated with positive attitude towards animals as well as concern for animal welfare. Hence, the present study highlighted the importance of considering ethical ideologies (idealism and relativism) of students regarding attitude toward wellbeing of animals and possible influence on future decision-making processes when it came to animal welfare.

**Abstract:**

Idealism and relativism are components of ethical ideologies which have been explored in relation to animal welfare and attitudes, and potential cultural differences. The present study investigated how ethical ideologies influenced attitude toward animals among undergraduate students. With the help of stratified random sampling, 450 participants were selected from both private and public sector universities in Pakistan. Research instruments consisted of a demographic sheet, the Ethics Position Questionnaire (EPQ), the Animal Attitude Scale—10-Item Version (AAS-10), and Animal Issue Scale (AIS). The study hypotheses were explored by employing various statistical analyses like Pearson Product Moment Correlation, independent sample t-test, ANOVA, and linear regression. Results revealed that there was a significant positive relationship between ethical ideologies (idealism and relativism) and attitude toward animals in students. Results further showed that students who consumed meat less frequently scored higher on relativism as compared to those who consumed meat more frequently (however, the effect size was small). It was also found that senior students held more idealistic ideologies as compared to freshman students. Finally, idealism positively predicted concern for animal welfare among students. The current study shed light on how ethical ideologies can shape and influence animal welfare. It further highlighted the potential cultural differences for the study variables by allowing for comparison with other published studies. By understanding these dynamics better, researchers will be better equipped to help students become informed citizens that may also influence future decision-making processes.

## 1. Introduction

The nature of human–animal interactions is diverse. Animals have been an essential part of human lives for centuries, from being hunting partners to companion animals at home and livestock raised for human consumption at farms. Given this bond and inter-dependence, significance of attitude toward animals and other related influential factors is increasingly being recognized within the field of human–animal relationships as well as animal welfare [1,2]. More recently, researchers have increasingly highlighted people’s positive attitude toward animals due to their numerous physical and psychological benefits for individuals as well as society. These benefits included enhanced physical health, increased happiness, reduced loneliness and anxiety, and enhanced social interactions [3,4,5].

Similarly, animals are an integral part of people’s lives in Pakistan. They serve various roles as pets, livestock, transportation and food animals, even being part of religious practices. However, despite playing such crucial roles in society, very little is understood about how people view and treat animals in general and how these views affect animal welfare. One variable that is pertinent to understanding attitude toward animals is understanding the effect of ethical ideologies. Forsyth (1980) highlights two aspects of ethical ideologies to explain differences in moral reasoning within his Ethics Position Questionnaire: idealism and relativism [6]. Individuals who hold idealistic ideologies firmly believe that positive outcomes always follow behaviors based on principles and ethics [6,7,8,9]. On the contrary, individuals with relativistic ideologies believe that decisions of moral nature should be taken while keeping in view certain rules and values prevalent in their respective society [6,7,8,9].

People holding idealistic view believe that their decisions always lead to positive outcomes and those who hold a relativistic approach believe that moral decisions are situational and based on local principles [8].

Individuals are further distinguished into four groups based on their level of idealistic and relativistic ideologies: (1) Individuals that hold high idealistic and relativistic ideologies are called situationists; (2) individuals with higher idealistic ideologies but lower relativistic ideologies are referred to as absolutists; (3) subjectivists possess lower idealistic and higher relativistic tendencies; (4) lastly, individuals who have both low idealistic and relativistic ideologies are known as exceptionists [6].

Individuals who were situationists believe that it is acceptable to not follow moral rules if it leads to better outcomes. Absolutists believe that rules of morality should be followed even if there are rewards which can be attained by not following them. Subjectivists are of the view that there will be unpreventable negative consequences for ethical decisions given that every person follows their own different moral principles. For exceptionists, whether an action is morally accurate or not depends on the outcomes it produces [8,10]. Individuals’ attitude towards animals is affected by the type of group they identify with. For example, absolutists view experiments involving animals as highly immoral, as compared to individuals belonging to other ethical ideology groups [11].

This idealistic or relativistic view toward ethical actions affects people’s views and attitude toward animals and animal welfare [8]. One study showed that individuals higher on idealism usually expressed more concern for animal use than those higher on relativism [9]. Given the strong moral principles reported by people holding idealistic tendencies, it will have a significant impact on welfare concerns for others as well as animals [12]. Further, it was found that younger people had more awareness regarding welfare of animals as compared to older people [13]. Considering this, analyzing and understanding students’ perception and ethical tendencies will have similar implications. Moreover, it is important for students to understand ethical issues and to implement a change that supports animal welfare. Young adults are at a crucial stage of transitioning into adulthood and have a vested interest in the future of the society. They are further exposed to a multitude of information through different channels that lead to formation of views and opinions about critical topics. To bring about such a change requires considering students’ existing understanding, including their views on moral reasoning and ethical concerns. This understanding, and consequently implementation for change, is crucial for promoting educational standards, including ‘decision-making competence’ in the future [14]. Hence, the present study aimed at understanding the ethical ideologies of students while raising awareness amongst said students about animal welfare concerns, which may consequently influence future decision making when it comes to ethical issues.

These moralistic tendencies were further influenced by other factors such as age, gender, religion, pet ownership, and geographic location [15,16,17], all of which were included and investigated in the present study. Women were found to be more concerned regarding animal welfare as compared to men [18]. Moreover, young adults exhibited more positive attitude toward animals as compared to middle-aged and older adults [13]. Another important consideration for comparison was the culture and society. Within developed countries, individuals were highly aware and concerned about the wellbeing of animals, and they formed their views regarding animals based on this awareness instead of analyzing animals in term of their respective advantages and disadvantages [19]. Studies in developed countries such as USA and the Netherlands showed significant impact of idealism, and not relativism, on attitude toward animals [20,21] indicating that relativism was not a critical factor in this respect. However, a study conducted in China showed how relativism had a negative relationship with individuals’ attitude toward animals [13].

As China is economically prospering so their focus of attention was on attaining the latest technological advancements due to which their awareness regarding animal welfare was highly limited. People in such countries formed their views regarding animals based on the advantages they can derive from them instead of considering their wellbeing. Given that Pakistan is a developing country with significant cultural differences from developed countries, the way human demographics interacted with ethical ideologies in forming attitudes towards animals will also differ. The current study aimed at highlighting any cultural influence on ethical ideologies by comparing the outcomes with other published studies.

### 1.1. Objectives

1. To investigate the relationship between ethical ideologies and attitude toward animals.
2. To investigate the effect of interaction of demographic variables (such as frequency of meat consumption and semester) in relation to ethical ideologies and attitude toward animals.
3. To investigate and highlight any cultural differences among the study variables.

### 1.2. Hypotheses

1. There will be a positive relationship among idealism, relativism, and attitude toward animals in students.
2. Students who score higher on idealism will report positive attitude toward animals.
3. Students who consume less meat will score higher on relativism.
4. Senior students will score higher on idealism as compared to freshman students.
5. Idealism will positively predict attitude toward animals in students.

## 2. Methodology

### 2.1. Research Design

The present study implemented a cross-sectional correlational research design. The study was cross-sectional in nature because the data were collected from research participants at one point in time in which the relationship between study variables was explored without any manipulation [22]. The study gained approval from the National Bioethics Committee (Ministry of National Health Services, Regulations and Coordination Islamabad, Government of Pakistan).

### 2.2. Instruments

The instruments used in the present study include a demographic sheet, Ethics Position Questionnaire (EPQ), the Animal Attitude Scale—10-Item Version (AAS-10), and Animal Issue Scale (AIS).

#### 2.2.1. Demographic Sheet

The demographics included were age, educational institution, degree program, semester, place of residence, religion/source of inspiration, degree of religiousness, pet ownership, type of pet, level of attachment with pet and frequency of meat consumption. Degree of religiousness was measured on a Likert-type question, ranging from “Not at all Religious” to “Highly Religious”. The level of attachment was measured using a 10-point rating scale, ranging from 0 (*Not at all Attached*) to 10 (*Highly Attached*). Level of comfort was measured similarly, ranging from 0 (*Low Comfort Level*) to 10 (*High Comfort Level*). Finally, frequency of meat consumption was measured on a 5-point Likert scale, from “Never” to “Every Day”.

#### 2.2.2. Ethics Position Questionnaire (EPQ) [6]

This instrument was used to determine the ethical ideology position held by individuals [23]. It is a 20 item self-report questionnaire on which respondents were asked to rate items on a 5-point Likert scale, ranging from 1 (*completely disagree*) to 5 (*completely agree*). It has two subscales: idealism and relativism. The idealism dimension asked respondents to give their responses on item such as “Risks to another should never be tolerated, irrespective of how small the risks might be” and “One should not perform an action which might in any way threaten the dignity and welfare of another individual”. The relativism dimension required respondents to express whether they agreed or disagreed with statements like “Moral standards should be seen as individualistic; what one person considers to be moral may be judged to be immoral by another person” and “What is ethical varies from one situation and society to another”. In the current study, Cronbach’s alpha of the scale is 0.90; for idealism subscale it is 0.88 and for relativism subscale it is 0.87.

#### 2.2.3. Animal Attitude Scale—10-Item Version (AAS-10) [15]

This questionnaire was used for measuring attitude toward animals. It is a self-report 20 item, 5-point Likert scale, ranging from 1 (*strongly disagree*) to 5 (*strongly agree*). Respondents were required to express their degree of agreement on items like “It is morally wrong to hunt an animal just for sport” and “It is unethical to breed purebred dogs for pets when millions of dogs are killed in animal shelter each year”. Items 2, 3, 4, 7 and 8 were reverse scored. The higher the score on the scale the greater the concern for animal welfare. In the current study, the Cronbach’s alpha was 0.86.

#### 2.2.4. Animal Issue Scale (AIS) [24]

This questionnaire was used for measuring attitude toward animals in respondents. It had a total of 43 items and respondents rated items on a 5-point Likert scale ranging from 1 (*extremely acceptable*) to 5 (*extremely unacceptable*). It further had eight sections which included use of animals, disrupting animal integrity, killing animals, compromising animal welfare, experimenting on animals, changing animals’ genotypes, animals and the environment (harming animals to protect the environment), and societal attitudes toward animals (harming animals for social purposes). Greater score on the scale indicated a higher concern for wellbeing of animals [25]. Respondents rated items such as “Inflicting pain, injury or disease on animals”, “Killing animals because they are not native to the area where they live” and “Destroying the habitat of endangered animal species”. A reliability of 0.94 was reported in the current study for this questionnaire.

### 2.3. Sample and Demographic Characteristics

With the help of random sampling, a total sample of 450 participants (men = 76, women = 374) within an age range of 18–36 years was collected from both public and private universities of Pakistan. The inclusion criteria for the present study pertained to any individual who was enrolled in an undergraduate program in a university and who was at least 18 years of age. For distribution of sample along demographic variables, see Table 1.

Table 1 illustrates the distribution of sample *(N =* 450) along various demographics. A majority of the participants were women (83%). The majority of students were doing their Bachelor of Science in psychology while the least number of students were enrolled in a Bachelor of Arts in psychology degree and Bachelor of Science in journalism degree. The highest frequency of student belonged to the 1st to 3rd semester. Most of the students resided in an urban section. The greatest number of students were moderately religious and did not own a pet. Finally, majority of the students consumed meat two to three times a week.

### 2.4. Procedure

For university sample selection, a list of accredited public and private universities in Pakistan for each province was obtained through the Higher Education Commission (HEC) website, and with the help of stratified random selection, three universities were selected from each province to be included in the sample. The purpose for this selection was to increase the representativeness of the sample. Afterwards, the responsible authorities were contacted for permission and their assistance requested for conducting the research while providing all the necessary information through the proper channels/protocols. In cases where institutions declined to participate in the research or there was a lack of response from a particular university within the specified amount of time, another university was selected from the remaining list with the help of stratified random selection until there was a balanced sample from each province.

Institutes assenting to participate in the study were requested to provide a list of their active departmental classes across all semesters and using a stratified random sampling technique, classes were selected for inclusion in the study. Based on the institutional protocol, we requested either the focal person or the instructor to distribute the survey questionnaires either in printed form or through an online link generated through Qualtrics software among participants. Any queries on the part of the focal person/instructor were cleared beforehand to optimize clear communication with the participants. The survey consisted of the demographic sheet, the Ethics Position Questionnaire (EPQ), the Animal Attitude Scale—10-Item Version (AAS-10), and Animal Issue Scale (AIS). The average time required for filling the survey was 15–20 min. Data were analyzed using appropriate statistical methods. With the help of latest version of SPSS Statistical software, the collected sample data were tested for normal distribution and where required, were translated into normal distribution. Levene test was used to determine homogeneity of the variances. Pearson Product Moment Correlation was computed for investigating the relationship among research study variables. Independent sample t-test and Analysis of variance (ANOVA) were performed to determine respondents’ ethical ideologies and demographics that may affect their attitudes toward animals. Simple and multiple linear regression were employed for relating participants’ responses in EPQ to their responses on AIS and AAS-10 to identify which variables determined attitude toward animals by employing the above-described model and utilizing an alpha value of 0.05 for variables to enter the model. The finding of the research study was shared with participants through their respective email address.

## 3. Results

The present study aimed at exploring attitude toward animals among university students and the possible influence of ethical ideologies on such attitudes. Cronbach alpha reliabilities for research instruments were computed (see Table 2).

Table 2 shows psychometric properties for the scales used in the study. All the scales and their respective subscales had satisfactory reliability ranging from 0.94 to 0.86.

### 3.1. Correlation among Ethical Ideologies (Idealism and Relativism), Attitude toward Animals, and Concern for Animal Welfare

For investigating relationship among research variables Pearson Product Moment Correlation was computed (see Table 3).

Table 3 shows a significantly stronger positive correlation between ethical position scale and the subscale idealism (*r* (448) = 0.85, *p* = 0.00). A significant positive correlation between ethical position scale and its respective subscale relativism (*r* (448) = 0.86, *p* = 0.00) was also found. Furthermore, results showed a significantly moderate positive relationship between ethical position (ethical ideologies) and attitude toward animals (*r* (448) = 0.38, *p* = 0.00). A significantly moderate positive relationship also existed for ethical position (ethical ideology) and concern for animal welfare (*r* (448) = 0.35, *p* = 0.00). Furthermore, idealism had a significantly moderate positive relationship with attitude toward animals (*r* (448) = 0.39, *p* = 0.00). A small significantly positive relationship was evident between relativism and attitude toward animals (*r* (448) = 0.25, *p* = 0.00). Moreover, a moderate significantly positive relationship was found between idealism and concern for animal welfare (*r* (448) = 0.43, *p* = 0.00). A small significantly positive relationship was found between relativism and concern for animal welfare (*r* (448) = 0.18, *p* = 0.00). Lastly, a stronger significantly positive relationship was found between attitude toward animals and concern for animal welfare (*r* (448) = 0.74, *p* = 0.00).

### 3.2. Difference among Ethical Ideologies, Attitude toward Animals, and Concern for Animal Welfare for Frequency of Meat Consumption in University Students

Independent sample *t*-test was used to study differences for ethical ideologies (idealism and relativism), animal attitude and concern for animal welfare between low level meat consumption and high level meat consumption groups (see Table 4).

Table 4 shows significant differences along frequency of meat consumption for ethical position and idealism. Results indicated that students consuming less amount of meat had a greater score on ethical position ideologies as compared to students who consumed more meat. However, the value of Cohen’s *d* was 0.22 (<0.50) which indicated small effect size. Furthermore, findings revealed that students consuming less meat held greater relative ideologies as compared to students who consumed more meat. The value of Cohen’s *d* was 0.24 (<0.50) which indicated small effect size. Nonsignificant findings were found for the remaining variables.

### 3.3. Difference along Semester/Stage of Program for Idealism in University Students

One-way ANOVA and post hoc analysis (Tukey Kramer for significant results only) were carried out to study the role of stage of the program/semester for idealism in university students (See Table 5).

Table 5 reveals that senior students held more idealistic ideologies as compared to freshman students. Furthermore, the value of η^2^ was 0.01 (<0.20) which showed small effect size. Post-hoc comparison indicated significant mean group differences for freshman group with senior group.

### 3.4. Predictive Role of Ethical Position on Attitude toward Animals

Simple linear regression was computed for investigating predictive role of ethical position on attitude toward animals in university students (see Table 6).

Table 6 show the impact of ethical position on attitude toward animals in university students. The *R*^2^ value of 0.38 revealed that the predictor, ethical position, explained 38% variance in outcome variable which was attitude toward animals with *F* (1, 448) = 75.3, *p* > 0.001. Findings indicated that ethical position positively predicted attitude toward animals (β = 0.38, *p* > 0.001).

### 3.5. Predictive Role of Ethical Position on Concern for Animal Welfare

Simple linear regression was computed for investigating predictive role of ethical position on concern for animal welfare in university students (see Table 7).

Table 7 shows impact of ethical position on concern for animal welfare in university students. The *R*^2^ value of 0.13 revealed that ethical position as a predictor explained 13% variance in concern for animal welfare which was the outcome variable with *F* (1, 448) = 64.7, *p* > 0.001. The results showed that ethical position positively predicted concern for animal welfare (β = 0.35, *p* > 0.001).

### 3.6. Predictive Role of Idealism and Relativism on Attitude toward Animals

Multiple linear regression was computed for investigating predictive role of idealism and relativism on attitude toward animals in university students (see Table 8).

Table 8 shows effect of idealism and relativism on attitude toward animals in university students. The *R*^2^ value of 0.16 showed that idealism and relativism explained 16% variance in the outcome variable which was attitude toward animals with *F* (2, 447) = 43.8, *p* < 0.001. The findings revealed that idealism positively predicted attitude toward animals (β = 0.36, *p* < 0.001) while nonsignificant results were found for relativism regarding attitude toward animals (β = 0.09, *p* > 0.05).

### 3.7. Predictive Role of Idealism and Relativism on Concern for Animal Welfare

Multiple linear regression was computed for investigating predictive role of idealism and relativism on concern for animal welfare in university students (see Table 9).

Table 9 shows impact of idealism and relativism on concern for animal welfare in university students. The *R*^2^ value of 0.19 showed that idealism and relativism explained 19% variance in the outcome variable that was concern for animal welfare with *F* (2, 447) = 52.3, *p* < 0.001. Results revealed that idealism positively predicted concern for animal welfare (β = 0.45, *p* < 0.001), whereas relativism did not significantly predict concern for animal welfare (β = −0.03, *p* > 0.05).

## 4. Discussion

The aim of the present study was to investigate how components of ethical ideologies (idealism and relativism) influenced attitude toward animals in the student population. Results supported the first hypothesis of the study that a significant positive relationship existed between idealism and attitude toward animals (see Table 3). This finding was in line with results of previous studies which showed that individuals holding idealistic ethical tendency had positive attitude towards animals, and they were more concerned with animal wellbeing [8,9,13,26]. People having an idealistic ideology were selflessly worried regarding the welfare of others without any cost–benefit analysis [12], so it was expected that such people showed excessive concern towards the wellbeing of animals as well. In addition, these individuals had a firm belief that harm could always be prevented, hence, they considered animal issues (like killing animals, keeping animals as pets, experimenting on animals, and such) very seriously while being concerned about animal welfare [12,27].

Hypothesis two of the study predicted there will be a significant positive relationship between relativism and attitude toward animals. The results confirmed this (see Table 3). This result was inconsistent with findings of previous studies highlighting a nonsignificant relationship between the two variables in the US [8,20]. Furthermore, this result was also inconsistent with findings from a study conducted in China which found a significant negative relationship between relativism and attitude toward animals [13]. This result may be accounted for by cultural differences. As Pakistan is an Islamic state and the religion promotes fair as well as humane treatment of all living things especially animals [28], people having relativistic ideologies hold a positive attitude towards welfare of animal due to their faith, even if they view animals in relation to the benefits they provide with situational factors in mind for moral decision making. Another explanation could be that Asian countries follow collectivist ideologies and universal principles are not that important for people residing in such countries [29,30]. Individuals living in Western countries follow individualist ideologies which focus on universal principles when it comes to interpreting various situations [29,30]. Hence, the relationship between relativism and attitude toward animals in students from Asian and Western countries could possibly differ.

The study outcomes further supported Hypothesis 3 which highlighted a significant difference along frequency of meat consumption for relativism among students (see Table 4). However, it should be noted that the effect size was small and as such, should be taken into consideration. One possible explanation is related to the price of meat. Certain meat products were perceived as luxury item in countries such as China and people consumed such items in a reduced amount [31] as they cost more. Pakistan being a developing country contains many people, especially students, belonging to lower-middle class and middle-middle class who cannot afford to eat luxury items on a regular basis [32]. An alternative explanation could be that students high on relativism were vegetarians who consumed no meat because of reasons like health or moral concerns while on the other hand very few students avoided meat because of being concerned with animal welfare [33].

The study results indicated a significant difference along stage of program (semester) for idealism among students (see Table 5). In support of these findings, a previous study indicated that students more progressed in their studies and at a later stage of education showed more concern towards harming animals [34]. This could be because students at more advanced stages of their education had more access to information regarding animal wellbeing as compared to students who were freshly admitted into a university [34]. Therefore, senior students developed greater idealistic tendencies regarding animal welfare as compared to students who were freshman, sophomore, or junior levels due to being more informed.

Lastly, idealism positively predicted favorable attitude toward animals in students (see Table 8). This result was consistent with findings of previous studies which showed that idealism had a positive association with attitude toward animals [8,20]. Individuals who believed that their ethical behaviors, based on universal principles, led to more desirable outcomes held favorable attitude towards animals and showed greater concern for their welfare [13].

### 4.1. Limitation and Suggestion

For the present study, all information was obtained from a self-reported questionnaire, resulting in the possibility of response bias. Including multiple assessments over certain times and deeper exploration through employing qualitative methods will provide a richer and thorough understanding of ethical ideologies and related factors. Since the survey was administered during the pandemic while universities were still adjusting to the transition, this may have impacted the responses. A consideration of the possible influences of changes within university dynamics due to the pandemic needs to be made. Furthermore, COVID-19 interfered with the data collection process by limiting access to the target sample. Due to this limitation, some of the research data was collected through online questionnaires while the remaining was collected in-person. There might have been an uncertainty in responses obtained from participants based on the mode of completing the research questionnaire. Another limitation of the study was that confounders were not accounted for during the analysis. The study outcomes showed a significant positive relationship of relativism with attitude toward animals which contradicted previous research findings. This indicated that the way relativism interacted with attitude toward animals and concern for animal welfare differed across developing countries, especially Muslim countries, and may require further investigation for cultural and religious differences. In addition, the unequal distribution of participants in demographic groups could have affected the research outcomes. Participants ought to be balanced across various human demographics to acquire a comprehensive understanding regarding the interaction of the study variables.

### 4.2. Implications

The present study highlighted the significance of ethical ideologies for influencing students’ attitude toward animals and concern for animal welfare. This is an important finding for future researchers who can further explore role of relativism in forming positive attitude towards animals in collectivist culture. The findings of the current study can be used to spread awareness regarding animal welfare and ways of improving concern among people regarding animal wellbeing through enhancing their ideological beliefs like idealism and relativism. In addition, relativism had a significant positive relationship with the frequency of meat consumption. Beliefs and culture of a country have a great impact on the consumption practices of its citizens so further research could be beneficial for exploring this domain, with findings of the current study being a starting point in this direction. More studies are required for further understanding the relationship between these and other variables within a collectivist, faith-based country.

## 5. Conclusions

To the best knowledge of the authors, the current study is the first one to explore ethical ideologies alongside attitude toward animals among students in Pakistan. The study showed that there was a positive relationship between ethical ideologies (idealism and relativism) and positive attitude toward animals. Individuals who believed that their moral behaviors always led to desirable outcomes as well as held universal moral principle were more concerned about animal welfare and held more positive attitudes towards animals. Further, individuals who believed that moral decision should be based on situational factors were also concerned for animal welfare. It was also evident from findings of study that students in advanced stages of their program held greater idealistic ideologies as compared to students in their initial semesters. Lastly, idealism was found to predict positive attitude and concern for animal wellbeing among students.

## Figures and Tables

**Table 1:** Frequencies and Percentages along Demographic Variables (N = 450).

Demographics	*f* (%)
Gender	
Men	76 (17)
Women	374 (83)
Degree program	
Advance diploma in clinical psychology	11 (2)
Bachelor of arts (psychology)	1 (0.2)
Bachelor of business administration	5 (1)
Bachelor of science (psychology)	382 (85)
Bachelor of science in computer	4 (1)
Bachelor of science (Journalism)	1 (0.2)
Master of science (psychology)	46 (10)
Semester	
1st–3rd	198 (44)
4th–6th	109 (24)
7th–8th	143 (32)
Place of residence	
Urban	374 (83)
Rural	76 (17)
Degree of religiousness	
Prefer not to say	46 (10)
Not religious at all	4 (0.9)
Slightly religious	42 (9)
Somewhat religious	39 (9)
Moderately religious	258 (57)
Highly religious	61 (14)
Own pet	
Yes	125 (28)
No	325 (72)
Amount of consumption of meat	
Never	14 (3)
Once a week or less	185 (41)
2–3 days a week	195 (43)
4–6 days a week	36 (8)
Everyday	20 (4)

**Table 2:** Psychometric properties for scales and their subscales (N = 450).

Scale	*M*	*SD*	Range	Cronbach’s α
Ethical Position Scale	80.5	10.8	30–100	0.90
Idealism Subscale	43.0	6.23	11–50	0.88
Relativism Subscale	37.5	6.35	12–50	0.87
Animal Attitude Scale	35.8	5.82	16–50	0.86
Animal Issue Scale	156.5	23.0	72–211	0.94

**Table 3:** Correlation among ethical position, idealism, relativism, attitude toward animals, and concern for animal welfare (N = 450).

	Variables	1	2	3	4	5
1	Ethical Position	-				
2	Idealism	0.85 **	-			
3	Relativism	0.86 **	0.46 **	-		
4	Attitude toward Animals	0.38 **	0.39 **	0.25 **	-	
5	Concern for Animal Welfare	0.35 **	0.43 **	0.18 **	0.74 **	-

** *p* < −0.01.

**Table 4:** Level of meat consumption along ethical position, relativism, idealism, attitude toward animals, and concern for animal welfare (N = 450).

	Low Level Meat Consumption		High Level Meat Consumption				
Variables	*M*	*SD*	*M*	*SD*	*t* (448)	*p*	Cohen’s *d*
Ethical Position	81.8	10.2	79.4	11.2	2.28	0.02	0.22
Idealism	43.4	5.67	42.7	6.64	1.33	0.19	
Relativism	38.3	6.14	36.8	6.45	2.57	0.01	0.24
Attitude toward Animals	36.2	6.96	35.6	5.71	1.11	0.27	
Concern for Animal Welfare	155.9	23.1	157.1	22.9	−0.55	0.58	

**Table 5:** Mean, standard deviation and one-way ANOVA in idealism across semesters (N = 450).

	Freshman		Sophomores and Juniors		Seniors				
Variables	*M*	*SD*	*M*	*SD*	*M*	*SD*	*F* (2, 447)	η^2^	Post-Hoc
Idealism	42.3	6.40	42.7	6.55	44.0	5.55	3.11 *	0.01	1 < 3

* *p* < 0.05.

**Table 6:** Regression coefficient of ethical position on animal attitude (N = 450).

Variable	*B*	β	*SE*
Constant	19.3 ***		1.92
Ethical position	0.20 ***	0.38	0.02
R^2^	0.38		

*** *p* < 0.001.

**Table 7:** Regression coefficient of ethical position on concern for animal welfare (N = 450).

Variable	*B*	β	*SE*
Constant	95.6 ***		7.64
Ethical position	0.76 ***	0.35	0.09
R^2^	0.13		

*** *p* < 0.001.

**Table 8:** Regression coefficient of idealism and relativism on attitude toward animals (N = 450).

Variables	*B*	*SE*	*t*	*p*	95% CI
Constant	18.5	1.91	9.67	0.00	[14.7, 22.3]
Idealism	0.33	0.05	7.33	0.00	[0.24, 0.42]
Relativism	0.08	0.04	1.77	0.08	[−0.01, 0.17]

*Note.* CI = Confidence Interval.

**Table 9:** Regression coefficient of idealism and relativism on concern for animal welfare (N = 450).

Variables	*B*	*SE*	*t*	*p*	95% CI
Constant	89.7	7.44	12.1	0.00	[75.1, 104.3]
Idealism	1.66	0.18	9.36	0.00	[1.31, 2.01]
Relativism	−0.12	0.17	−0.69	0.49	[−0.46, 0.22]

*Note.* CI = Confidence Interval.

## Data Availability

The data presented in this study are available from the corresponding author upon request.
